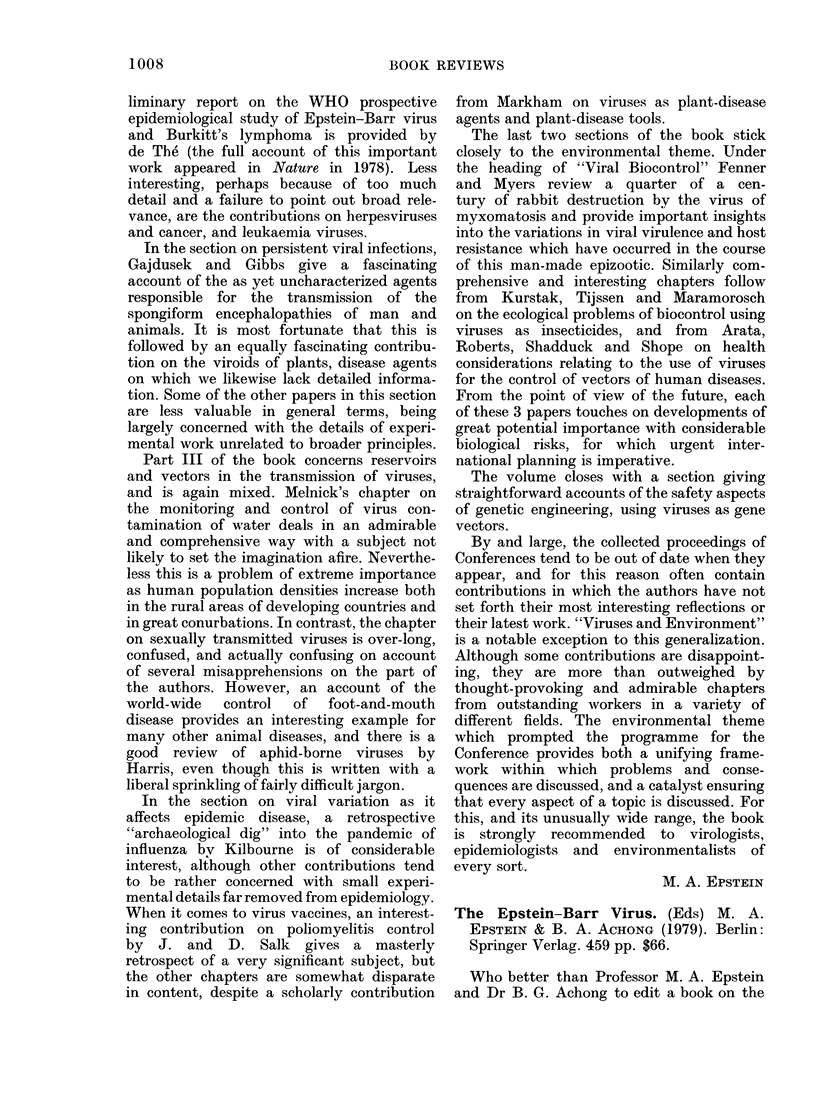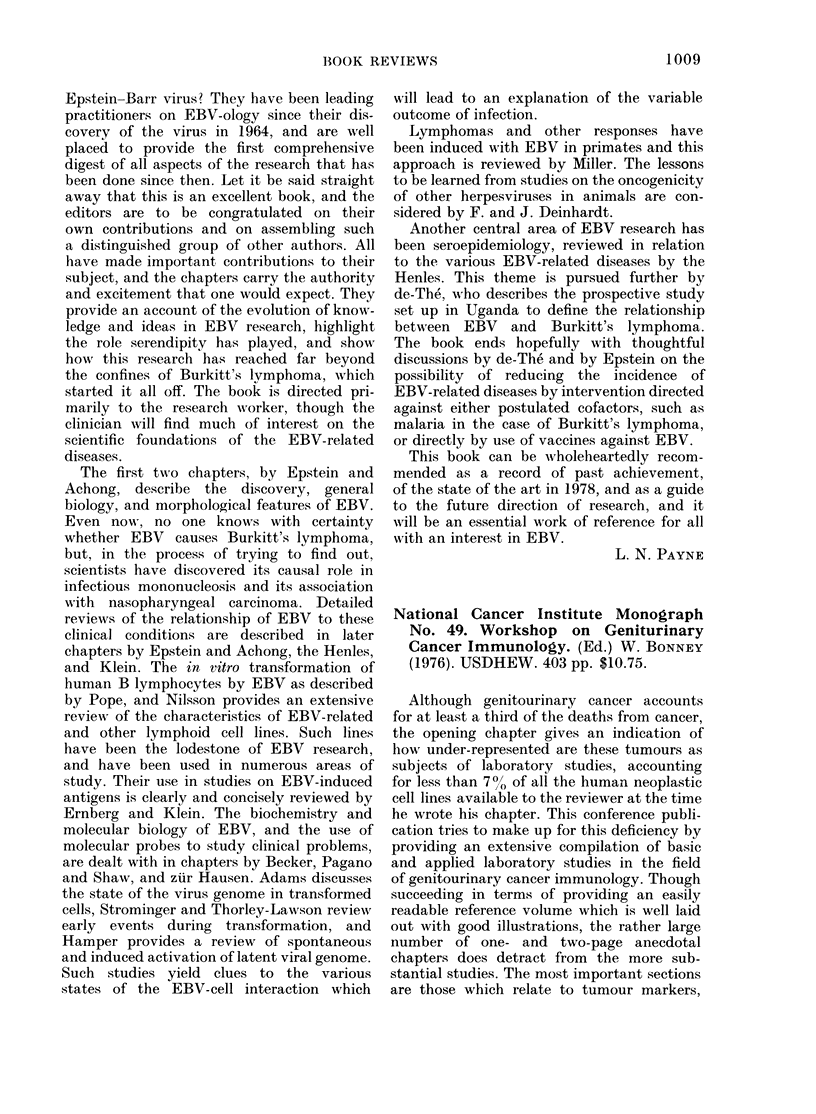# The Epstein-Barr Virus

**Published:** 1980-06

**Authors:** L. N. Payne


M. A. Epstein

**The Epstein–Barr Virus.** (Eds) M. A. Epstein & B. A. Achong (1979). Berlin: Springer Verlag. 459 pp. $66.

Who better than Professor M. A. Epstein and Dr B. G. Achong to edit a book on the

Epstein–Barr virus? They have been leading practitioners on EBV-ology since their discovery of the virus in 1964, and are well placed to provide the first comprehensive digest of all aspects of the research that has been done since then. Let it be said straight away that this is an excellent book, and the editors are to be congratulated on their own contributions and on assembling such a distinguished group of other authors. All have made important contributions to their subject, and the chapters carry the authority and excitement that one would expect. They provide an account of the evolution of knowledge and ideas in EBV research, highlight the role serendipity has played, and show how this research has reached far beyond the confines of Burkitt's lymphoma, which started it all off. The book is directed primarily to the research worker, though the clinician will find much of interest on the scientific foundations of the EBV-related diseases.

The first two chapters, by Epstein and Achong, describe the discovery, general biology, and morphological features of EBV. Even now, no one knows with certainty whether EBV causes Burkitt's lymphoma, but, in the process of trying to find out, scientists have discovered its causal role in infectious mononucleosis and its association with nasopharyngeal carcinoma. Detailed reviews of the relationship of EBV to these clinical conditions are described in later chapters by Epstein and Achong, the Henles, and Klein. The *in vitro* transformation of human B lymphocytes by EBV as described by Pope, and Nilsson provides an extensive review of the characteristics of EBV-related and other lymphoid cell lines. Such lines have been the lodestone of EBV research, and have been used in numerous areas of study. Their use in studies on EBV-induced antigens is clearly and concisely reviewed by Ernberg and Klein. The biochemistry and molecular biology of EBV, and the use of molecular probes to study clinical problems, are dealt with in chapters by Becker, Pagano and Shaw, and zür Hausen. Adams discusses the state of the virus genome in transformed cells, Strominger and Thorley-Lawson review early events during transformation, and Hamper provides a review of spontaneous and induced activation of latent viral genome. Such studies yield clues to the various states of the EBV-cell interaction which will lead to an explanation of the variable outcome of infection.

Lymphomas and other responses have been induced with EBV in primates and this approach is reviewed by Miller. The lessons to be learned from studies on the oncogenicity of other herpesviruses in animals are considered by F. and J. Deinhardt.

Another central area of EBV research has been seroepidemiology, reviewed in relation to the various EBV-related diseases by the Henles. This theme is pursued further by de-Thé, who describes the prospective study set up in Uganda to define the relationship between EBV and Burkitt's lymphoma. The book ends hopefully with thoughtful discussions by de-Thé and by Epstein on the possibility of reducing the incidence of EBV-related diseases by intervention directed against either postulated cofactors, such as malaria in the case of Burkitt's lymphoma, or directly by use of vaccines against EBV.

This book can be wholeheartedly recommended as a record of past achievement, of the state of the art in 1978, and as a guide to the future direction of research, and it will be an essential work of reference for all with an interest in EBV.

L. N. PAYNE